# Gatekeeping astrocyte identity

**DOI:** 10.7554/eLife.80232

**Published:** 2022-06-20

**Authors:** Alexis Cooper, Benedikt Berninger

**Affiliations:** 1 Centre for Developmental Neurobiology, King's College London London United Kingdom; 2 The Francis Crick Institute London United Kingdom; 3 University Medical Center of the Johannes Gutenberg University Mainz Germany

**Keywords:** PTBP1, astrocyte-to-neuron conversion, lineage reprogramming, parkinson's disease, brain repair, astrocyte, Mouse

## Abstract

New findings cast doubt on whether suppressing the RNA-binding protein PTBP1 can force astrocytes to become dopaminergic neurons.

**Related research article** Chen W, Zheng Q, Huang Q, Ma S, Li M. 2022. Repressing PTBP1 fails to convert reactive astrocytes to dopaminergic neurons in a 6-hydroxydopamine mouse model of Parkinson’s disease. eLife **11**:e75636. doi: 10.7554/eLife.75636.

As a cell acquires its final identity, it usually closes the door on the other cellular fates it could have adopted. Valiant guardian mechanisms ensure that this gate remains shut, even as a plethora of signals threaten to crack it open once more. These processes are particularly crucial for cells from highly related lineages – such as astrocytes and neurons, two types of brain cells that derive from the same progenitor cells.

In the past 15 years, work has shown that astrocytes, which belong to a class of non-neuronal cells that support and fine-tune the activity of nerve cells, can be converted into neurons if they are forced to express neurogenic transcription factors (Götz and Bocchi, 2021). In some brain regions such as the mouse striatum, injury can even re-activate a neurogenic programme which is otherwise suppressed (Magnusson et al., 2014). This raises the intriguing possibility that specific mechanisms help to safeguard the identity of astrocytes; these processes could also be harnessed to awaken neurogenic potential in astrocytes and help repair neural damage.

A top-selling candidate for gatekeeping the astrocyte-to-neuron conversion is the RNA-binding PTBP1 which, in vitro, inhibits neuronal fate by ensuring that a master repressor of neuronal genes remains active (Makeyev et al., 2007). The discovery of these regulatory interactions suggested that simply downregulating PTBP1 could release the brake on a neurogenic programme in non-neuronal cells (Xue et al., 2013; Figure 1A). In fact, two recent bodies of work suggest that when PTBP1 is knocked down, mouse astrocytes can turn into dopaminergic neurons with remarkable efficiency (Qian et al., 2020, Zhou et al., 2020). This class of nerve cells degenerates in Parkinson’s disease, and both studies reported a drastic amelioration of motor deficits in a mouse model of this condition, with enormous implications for new brain therapies. Now, in eLife, Mingtao Li and colleagues at Sun Yat-sen University – including Weizhao Chen as first author – report results that question these findings (Chen et al., 2022).

**Figure 1.**
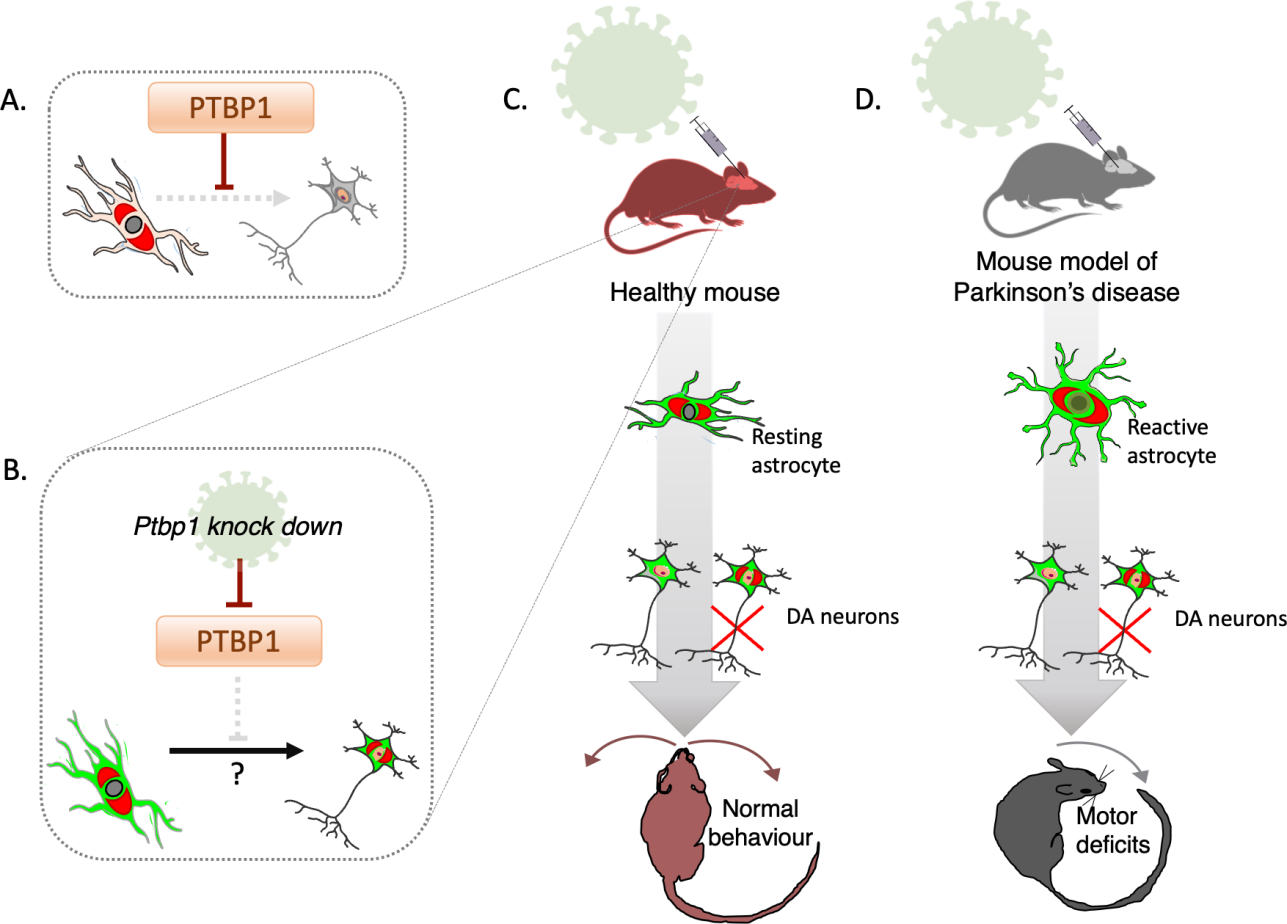
Deactivating PTBP1 in astrocytes fails to convert the cells into neurons. (**A**) The hypothesis tested by Chen et al. is that the protein PTBP1 stops astrocytes (pale pink) from becoming neurons (grey). (**B**) Adeno-associated viruses (green) can deliver the genetic information necessary to deactivate PTBP1 in astrocytes which have been genetically tagged (red). This potentially allows the cells to be converted into dopaminergic neurons which still carry the tag (red) reflecting that they have originated from astrocytes, as well as the viral label (green). (**C**) Chen et al. used a mouse model in which astrocyte origin could be traced to investigate whether knocking down PTBP1 using adeno-associated viruses may lead to the cells becoming dopaminergic neurons (DA carrying the red tag); this turned out not to be the case (red cross). Instead, dopaminergic neurons carrying the viral signal (green-only cells) were identified, which did not originate from astrocytes. (**D**) Similar experiments were conducted in a mouse model of Parkinson’s disease. In this instance, a toxin was introduced to kill endogenous dopaminergic neurons and render resting astrocytes reactive; in this injury-triggered state, astrocytes exhibit features associated with stem cells. Deactivating PTBP1 in reactive astrocytes again failed to turn them into neurons, and the mice still exhibited motor deficits.

The team focused on whether the seemingly converted dopaminergic cells truly derived from astrocytes, using a transgenic mouse line that faithfully reports the origin of astrocytes. PTBP1 was successfully knocked down in astrocytes by using adeno-associated viruses, viral vectors that contain the information necessary to suppress the protein only in astrocytes (Figure 1B).

Dopaminergic neurons carrying the viral label were identified, but to the team’s surprise, none of these were positive for the genetic tag that marked astrocyte origin (Figure 1C). This data strongly suggests that these neurons have not emerged from converted astrocytes, but that instead the viral vectors had lost their original specificity. This adds to recent, baffling observations, which highlighted that adeno-associated viruses designed to activate the expression of neuronal conversion factors only in astrocytes, fail to attain the required specificity (Wang et al., 2021).

Chen et al. then checked whether the manipulation could be successful if done on a type of astrocyte that may be more prone to changing its identity. These ‘reactive’ astrocytes emerge in damaged tissues, where they start to display traits present in neural stem cells (Sirko et al., 2013). A toxin was used to kill dopaminergic neurons as PTBP1 was suppressed in reactive astrocytes, yet even this scenario failed to turn the cells into neurons (Figure 1D). Conducting this manipulation in a mouse model of Parkinson’s disease also did not lead to improvements in the animals’ motor deficits. How this negative finding can be reconciled with earlier studies showing that these symptoms were corrected upon PTBP1 deactivation will require further clarification.

Taken together, the work by Chen et al. strongly argues against PTBP1 being the sole gatekeeper between astrocyte and neuronal fates, while also stressing the importance of rigorous genetic lineage tracing when conducting in vivo reprogramming. Still, given the powerful control that PTBP1 exerts on the molecular switch that represses neuronal genes, it would be premature to fully move away from studying the impact of this protein on astrocyte identity and function.
